# Gray matter differences associated with menopausal hormone therapy in menopausal women: a DARTEL-based VBM study

**DOI:** 10.1038/s41598-023-28673-2

**Published:** 2023-01-25

**Authors:** Tae-Hoon Kim, ByoungRyun Kim, Youe Ree Kim, Chang-Won Jeong, Young Hwan Lee

**Affiliations:** 1 Medical Convergence Research Center, Wonkwang University and Hospital, Iksan, 54538 Republic of Korea; 2 Department of Obstetrics and Gynecology, Wonkwang University Hospital, Iksan, 54538 Republic of Korea; 3 Department of Radiology, Wonkwang University School of Medicine, Iksan, 54538 Republic of Korea

**Keywords:** Health care, Medical imaging, Magnetic resonance imaging

## Abstract

Menopausal hormone therapy (MHT) in women can reduce troublesome menopause symptoms and prevent cognitive decline. This cross-sectional study investigated the MHT-related effect on brain morphology and its association with sex hormones in menopausal women by using an optimized diffeomorphic anatomical registration through exponentiated Lie algebra (DARTEL)-based voxel-based morphometry (VBM) method. Twenty-one menopausal women without MHT (noMHT) and 20 menopausal women with MHT were included in this study. Magnetic resonance imaging data were processed using SPM 12 with DARTEL-based VBM whole brain analysis approach. A 2-sample t-test and analysis of covariance (ANCOVA) adjusting for age and total intracranial volume were used to compare GM volume between noMHT and MHT women. The association between MHT (treatment period, hormones levels) and brain volume variations were analyzed by Spearman correlation. MHT women showed significantly larger volumes of the superior/middle/inferior frontal gyri, hypothalamus, inferior temporal gyrus, parahippocampal gyrus, hippocampus, cerebellar cortex, postcentral gyrus, precuneus, angular gyrus, supplementary motor area, superior occipital gyrus, and precentral gyrus compared to the noMHT women. The volumes of the angular gyrus and hypothalamus in MHT women positively correlated with treatment period. On the other hand, the hypothalamic volume negatively correlated with FSH and LH levels, and the volumes of the inferior frontal gyrus, and angular gyrus negatively correlated with progesterone levels, respectively. MHT-treated women showed larger GM volume than noMHT women. The anatomical structures that showed greater volume in association with MHT included the deep brain areas, frontal, temporal, parietal, and occipital gyri.

## Introduction

Menopause is a natural biophysiological event associated with an age-related reduction in hormone secretion that results in follicular recession^1,2^. Menopausal symptoms and signs affect the central nervous system, genitourinary tract, and musculoskeletal system in approximately 80% of women, of which 20% are severely affected^3,4^. The physiological changes in the duration of menopause exert a wide variety of effects on brain morphology and development and cognitive function^5,6^. Post-menopausal women have frequently suffered cognitive dysfunction (memory loss and cognitive difficulties) and a higher risk of Alzheimer’s disease compared to men of the same age^4,7^. Therefore, investigating the hormonal effects on the brain in women provides a specific opportunity to examine menopause-related alterations.

Several studies have investigated the impact of sex hormones on cerebral structure during the menstrual cycle^8–11^ or oral contraceptive intake^12–14^. The gray matter (GM) volumes in the regions including of the amygdala, hippocampus, temporal gyrus and parietal gyrus fluctuated by sex hormones across the menstrual cycle^8–11^. Some studies reported that women taking oral contraception decreased the cortical thickness of localized regions including of the anterior cingulate cortex, insula, and orbitofrontal gyrus, and decreased GM volumes in the amygdala (left side) and parahippocampal gyrus (anterior part)^12,13^. Other studies reported that oral contraceptive women group increased GM volumes in the regions of the cerebellum, fusiform gyrus, parahippocampal gyrus, temporal gyrus and parietal gyrus during the follicular phase and luteal phase, compared to healthy control women^13,14^. Also, several studies examined the relationship between sex steroid hormones and GM volume. A study found larger volume of the supplementary motor area (MOs), inferior frontal gyrus (IFG), olfactory cortex, and superior temporal gyrus (STG) in premenopausal women compared to postmenopausal women^6^. The study demonstrated that serum estradiol level positively correlated with GM volumes of the MOs, IFG, and STG, and follicle-stimulating hormone (FSH) negatively correlated with GM volumes of the MOs, IFG, and STG^6^. A longitudinal study^15^ reported that cerebral hippocampal volumes changed before and after hormone intake. Three different estrogen-dose groups (placebo, daily 1 mg, and daily 2 mg) were administered to healthy postmenopausal women for 3 months, and the bilateral hippocampal volume dose-dependently increased following 3 months of treatment^15^. Taking all of these findings into the consideration, the localized brain regions can be helpful for assessing the hormone therapeutic effects in conjunction with levels of sex steroid hormones.

Menopausal hormone therapy (MHT) medication can help reduce troublesome menopausal symptoms such as cognitive changes, mood changes, sleep problems, and sexual problems^16,17^. Several studies reported that MHT can reduce the incidence of Alzheimer’s disease by more than 64% and prevent age-related cognitive decline, proving that it has been used in the past or for a sufficient period of time^18,19^. A recent nationwide case–control study suggested a small increase in the absolute risk of Alzheimer’s disease associated with long-term (> 10 years) exposure to systemic MHT^20^, but this finding requires more corroborative evidence worldwide. Although this evidence does not support the use of MHT singularly for the primary prevention of chronic diseases, such as cardiovascular disease, osteoporosis, and cognitive impairment^3,21^, symptomatic women who begin MHT in early menopause may gain protection from certain chronic conditions^22,23^. However, it is still unclear how to affect menopausal symptoms and to protect the brain of the menopausal woman from declining.

With the recent neuroimaging technique, Goto et al*.*^24^ unveiled the acceleration of volume reduction with aging and menopause in the hippocampus of menopausal women using voxel-based morphometry (VBM) and atlas-based analysis methods. Menopausal estrogen therapy (ET) improves the structural integrity of tissue, inducing neuronal growth and similar trophic effects. Several morphometric studies^25–27^ reported that post-menopausal women who received the ET showed enhanced cognitive function and brain volume. A previous VBM study^28^ showed that post-menopausal women receiving ET had larger cortical GM volumes than post-menopausal women without ET, especially in the hippocampus-amygdala complex and cerebral cortex. Lord et al*.*^29^ reported larger hippocampal volumes in patients who received ET using a volumetric measurement method. A similar study^30^ suggested that ET is related to the retention of GM volume in the frontal, parietal, and temporal cortices in post-menopausal women. A correlative study^31^ reported a positive association between ET use and GM density in the inferior parietal lobules and the precuneus. In recent years, a VBM technique using diffeomorphic anatomical registration through an exponentiated Lie algebra (DARTEL) algorithm has grown in popularity due to its more accurate intersubject alignment for brain segmentation and registration than earlier VBM techniques^6,32^. DARTEL-based VBM of the whole cerebral cortex and menopause-associated areas (e.g., the pituitary gland, hippocampus and amygdala formation) will provide more accurate and statistically powerful information on cerebral volume changes related specifically to the effects of MHT in menopausal women. There have been few DARTEL-based VBM studies assessing the association between brain volumes and sex hormone levels in menopausal women^6^. The DARTEL VBM study reported that the localized GM volumes of the SMA, IFG, and SFG were positively correlated with the serum estradiol levels within the pre-menopausal and menopausal women, and among the area volumes, the SMA volume was negatively correlated with the duration of time after natural menopause in menopausal women. Until now, there was no study focusing on the associations between specific hormone levels and/or the length of menopausal treatment period.

For this study, we investigated the difference in GM between menopausal women with MHT and without MHT using a DARTEL-based VBM method and assessed the association between regional morphologic variation and MHT (including hormone levels and period of treatment).

## Results

### Demographic characteristics

This study compared MHT women with age-matched and body mass index (BMI)-matched menopausal women without MHT (noMHT group). Comparison of mean sex hormone levels and hormone therapeutic information in the two groups is shown in Table 1. In the MHT women group, the mean period of MHT was 6.2 ± 6.4 years (range, 0.5–21.1 years) on average. The estradiol level in MHT women was higher than that of the noMHT group (*p* < 0.001), while the levels of FSH (*p* = 0.029) and anti-müllerian hormone (AMH) (*p* = 0.047) were lower. These sex hormonal levels were significantly different between the two groups.

**Table 1:** Patient characteristics and sex hormone levels in the menopausal hormone therapy (MHT) and noMHT groups.

Hormone levels	noMHT (n = 21)	MHT (n = 20)	*p*-value*	Effect size *|*Cohen’s d*|*	Achieved power (input d, α = 0.05)	*N* need (each group) for 80% power
Age (year)	56.6 ± 3.8	57.2 ± 4.2	0.728	0.147	0.074	728
BMI (kg/m^2^)	23.7 ± 2.9	22.8 ± 2.4	0.368	0.029	0.051	18,667
Age of menopause (year)	50.3 ± 4.8	50.7 ± 3.3	0.742	–	–	–
MHT drug (drug name) (n)	–	Premina 10, Angeliq 6, Livial 2, Progynova 1, Duavive 1	–	–	–	–
MHT period (year, range) (min.–max.)	–	–	–	–	–	–
Anti-müllerian hormone^a^ [ng/mL]	0.0105 ± 0.002	0.0100 ± 0.000	0.047	0.360	0.203	123
Estradiol^b^ [pg/mL]	8.03 ± 1.83	28.02 ± 5.62	< 0.001	1.074	0.918	15
Testosterone^c^ [ng/mL]	0.13 ± 0.03	0.16 ± 0.03	0.779	0.211	0.101	354
Progesterone^d^ [ng/mL]	0.59 ± 0.54	0.06 ± 0.01	0.051	0.355	0.198	126
FSH^e^ [mIU/L]	67.20 ± 7.65	42.90 ± 3.90	0.029	0.859	0.765	23
LH^f^ [mIU/L]	33.39 ± 3.49	31.37 ± 2.71	0.233	0.143	0.073	769

Sex hormone data presented mean ± SEM. BMI: body mass index. MHT stands for menopausal hormone therapy. follicle-stimulating hormone: FSH; and luteinizing hormone: LH.

Cohen’s d was calculated for standardized effect size for measuring the difference between two group means. Refer to effect sizes as small (d = 0.2), medium (d = 0.5), large (d = 0.8).

Reference ranges for sex steroid hormones in pre-menopausal and post-menopausal women.

*The difference between noMHT and MHT groups was analyzed by independent two sample *t*-test.

^a^High > 4.0 ng/mL; normal 1.5–4.0 ng/mL; low normal 1.0–1.5 ng/mL; low 0.5–1.0 ng/mL; very low < 0.5 ng/mL.

^b^Pre-menopausal women, 11–526 pg/mL; post-menopausal women, less than 37 pg/mL.

^c^20–49 years old, 0.08–0.48 ng/mL; ≥ 50 years old, 0.03–0.41 ng/mL.

^d^Pre-menopause, follicular: 0.06–0.89, ovulation: 0.12–12.00, luteal: 1.83–23.90; postmenopause, < 0.05–0.13 ng/mL.

^e^Pre-menopausal women, 1.5–33.4 mIU/mL; post-menopausal women, 23–116.3 mIU/mL.

^f^Pre-menopausal women, 0.5–73.6 mIU/mL; post-menopausal women, 15.9–54.0 mIU/mL.

### Localized differences in brain volume in MHT

The total intracranial volumes (TIV) in the two groups are listed in Table 2. The GM volumes in noMHT and MHT women were 599.4 ± 46.8 mL and 587.2 ± 54.3 mL (*p* = 0.442), respectively, and the white matter (WM) volumes were 450.0 ± 56.6 mL and 443.7 ± 34.0 mL (*p* = 0.672), respectively. Cerebrospinal fluid (CSF) volumes of noMHT and MHT women were 473.1 ± 142.9 mL and 425.0 ± 157.3 mL, respectively (*p* = 0.311); and TIVs were 1522.7 ± 175.8 mL and 1456.0 ± 220.8 mL, respectively (*p* = 0.290). However, all tissue volumes and TIV were not significantly different.

**Table 2:** Differential intracranial brain volumes in noMHT and MHT women.

Tissues	Group			*p*-value^‡^
	NoMHT women (n = 21, A)	MHT women (n = 20, B)	Difference (A − B)	
GM^a^ (mL, CV^b^ %)	599.4 ± 46.8 (7.8)	587.2 ± 54.3 (9.2)	12.2	0.442
WM^a^ (mL)	450.0 ± 56.6 (12.6)	443.7 ± 34.0 (7.7)	6.3	0.672
CSF^a^ (mL)	473.1 ± 142.9 (30.2)	425.0 ± 157.3 (37.0)	48.1	0.311
GM^a^ + WM^a^ (mL)	1049.4 ± 93.2 (8.9)	1030.9 ± 81.3 (9.2)	18.5	0.502
Total volume (mL)	1522.7 ± 175.8 (11.5)	1456.0 ± 220.8 (15.2)	66.7	0.290

*MHT* menopausal hormone therapy, *GM* gray matter, *WM* white matter, *CSF* cerebrospinal fluid.

^‡^The difference between noMHT and MHT women was analyzed with independent two sample *t*-test.

^a^The volume was measured by using 'get_totals.m' function from segmented individual data on SPM12.

^b^The percentage indicates the coefficient of variation ( CV = [SD/volume mean] × 100).

**Figure 1:**
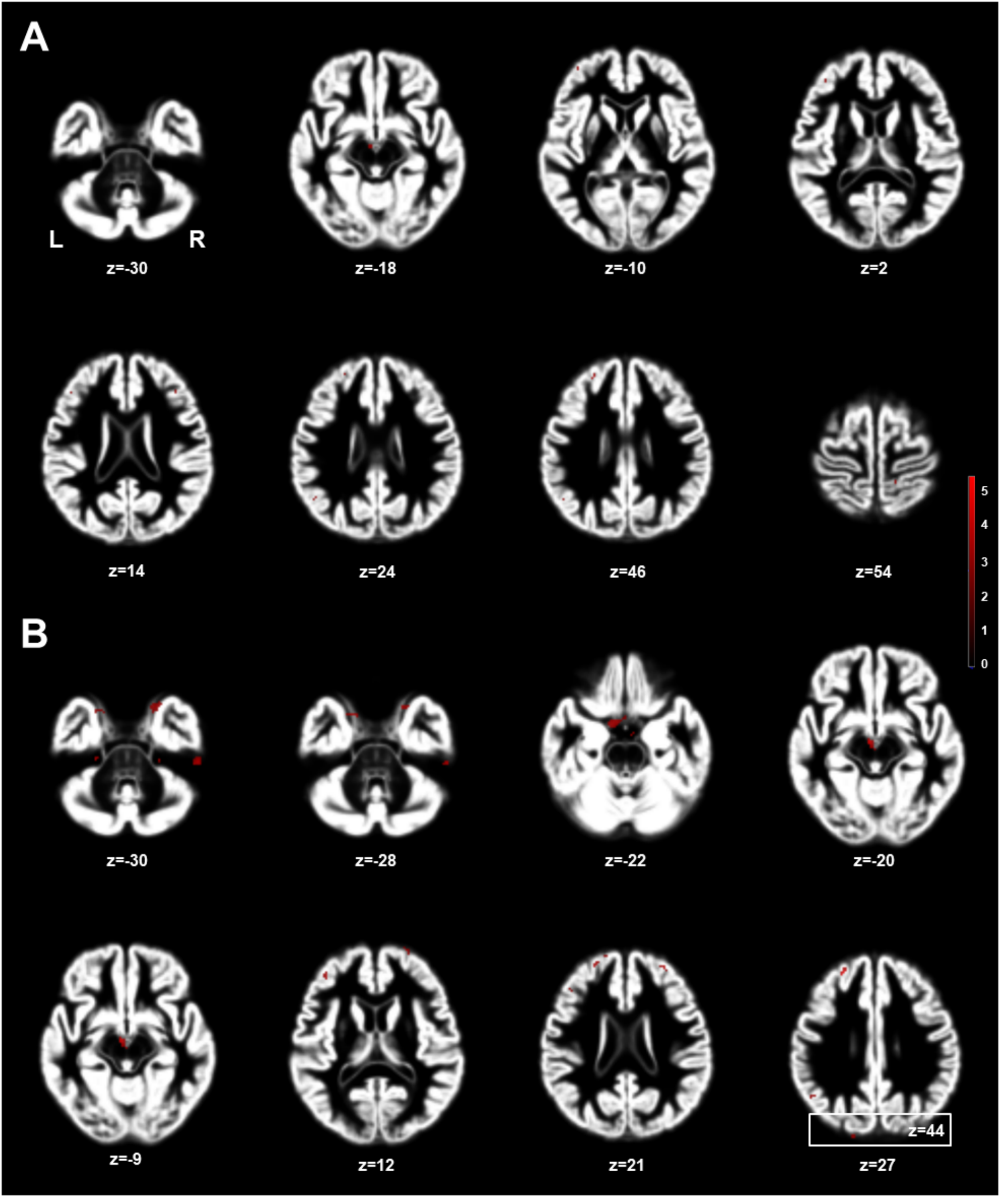
Brain areas with larger gray matter volumes in menopausal hormone therapy (MHT) women relative to noMHT women, which analyzed with independent two-sample *t*-test (**A**) and analysis of covariance (ANCOVA) (**B**) adjusting for age and total intracranial volume (TIV) (corrected *p* < 0.05; threshold *t* = 3.0, cluster size 50). The red color-coded pixels were scaled to the range (*t*-value 3.00–5.50) more than the cut-off threshold. L = left; R = right.

**Figure 2:**
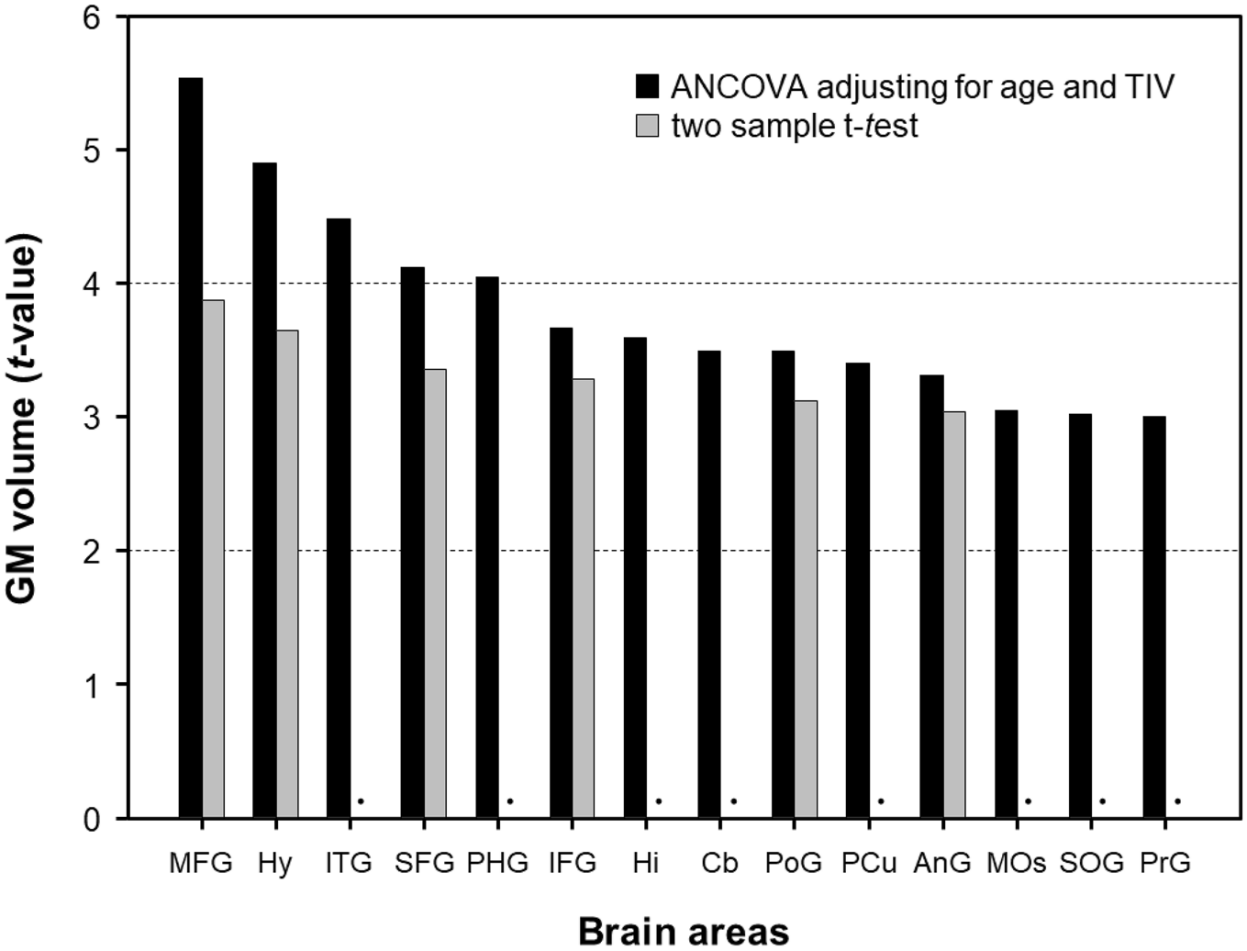
Gray matter volume (maximum *t*-value) observed in menopausal hormone therapy (MHT) women relative to noMHT group: independent two-sample *t*-test and analysis of covariance (ANCOVA) adjusting for age and total intracranial volume (TIV) (*p* < 0.05). Angular gyrus: AnG; Cerebellar cortex: Cb; Hippocampus: Hi; Hypothalamus: Hy; Inferior frontal gyrus: IFG; Inferior temporal gyrus: ITG; Middle frontal gyrus: MFG; Parahippocampal gyrus: PHG; Postcentral gyrus: PoG; Precentral gyrus: PrG; Precuneus: PCu; Superior frontal gyrus: SFG; Superior occipital gyrus: SOG; Supplementary motor area: MOs.

Figure 1 shows the significantly larger volumes of GM in the MHT group (cut-off *t*-value, 3.00; cluster size > 50) using DARTEL analysis, and the anatomical areas are summarized in Table 3. Using two-sample *t*-test (Fig. 1A), VBM indicated that MHT was associated with larger GM volumes in the MHT group, which included the middle frontal gyrus (MFG), hypothalamus (Hy), superior frontal gyrus (SFG), IFG, postcentral gyrus (PoG), and angular gyrus (AnG). With ANCOVA analysis adjusting for age and TIV (Fig. 1B), MHT-treated women showed larger GM volumes in the SFG/MFG/IFG, Hy, inferior temporal gyrus (ITG), parahippocampal gyrus (PHG), hippocampus (Hi), cerebellar cortex (Cb), PoG, precuneus (PCu), AnG, MOs, superior occipital gyrus (SOG), and pre-central gyrus (PrG), compared noMHT women. Figure 2 shows a bar graph in comparison of *t*-values using two different statistical analyses. The *t*-values by ANCOVA analysis were higher values than those using two sample *t*-test. However, there was no greater GM volume in the noMHT group.

**Table 3:** Larger GM volume areas in MHT group (*p* < 0.05; threshold *t*-value 3.0 and cluster size more than 50 pixels).

Anatomical area	Abbr	*t*-value	Equivalent z-value	MNI coordinate			No. of voxels
				x	y	z	
Two sample *t*-test							
Middle frontal gyrus	MFG	3.87	3.54	− 23	34	27	2373
Hypothalamus	Hy	3.64	3.36	− 5	− 14	− 9	3060
Superior frontal gyrus	SFG	3.35	3.12	11	42	23	2167
Inferior frontal gyrus	IFG	3.28	3.06	− 40	24	21	1269
Postcentral gyrus	PoG	3.12	2.93	15	− 41	54	147
Angular gyrus	AnG	3.04	2.86	− 45	− 54	25	613
ANCOVA adjusting for age and TIV							
Middle frontal gyrus	MFG	5.53	4.66	− 22	36	26	2966
Hypothalamus	Hy	4.89	4.24	− 4	− 14	− 9	292
Inferior temporal gyrus	ITG	4.48	3.96	52	− 28	− 30	797
Superior frontal gyrus	SFG	4.11	3.69	− 17	10	54	160
Parahippocampal gyrus	PHG	4.04	3.64	− 8	− 1	− 21	81
Inferior frontal gyrus	IFG	3.66	3.35	− 36	34	12	1179
Hippocampus	Hi	3.59	3.29	22	12	− 30	75
Cerebellar cortex	Cb	3.49	3.21	25	− 28	− 31	441
Postcentral gyrus	PoG	3.49	3.21	20	− 33	67	421
Precuneus	PCu	3.40	3.14	− 17	− 75	45	606
Angular gyrus	AnG	3.31	3.08	− 46	− 55	33	54
Supplementary motor area	MOs	3.04	2.86	− 9	15	52	108
Superior occipital gyrus	SOG	3.02	2.83	− 19	− 75	43	108
Precentral gyrus	PrG	3.00	2.81	30	− 24	54	130

*MNI* Montreal Neurological Institute, *ANCOVA* analysis of covariance, *TIV* total intracranial volume.

Moderate effect size of Cohen’s d (0.5) is threshold *t*-value 3.0.

*t*-value was calculated by two sample *t*-test and ANCOVA with adjusting covariates of age and TIV.

### Correlation between localized brain volume and MHT

**Figure 3:**
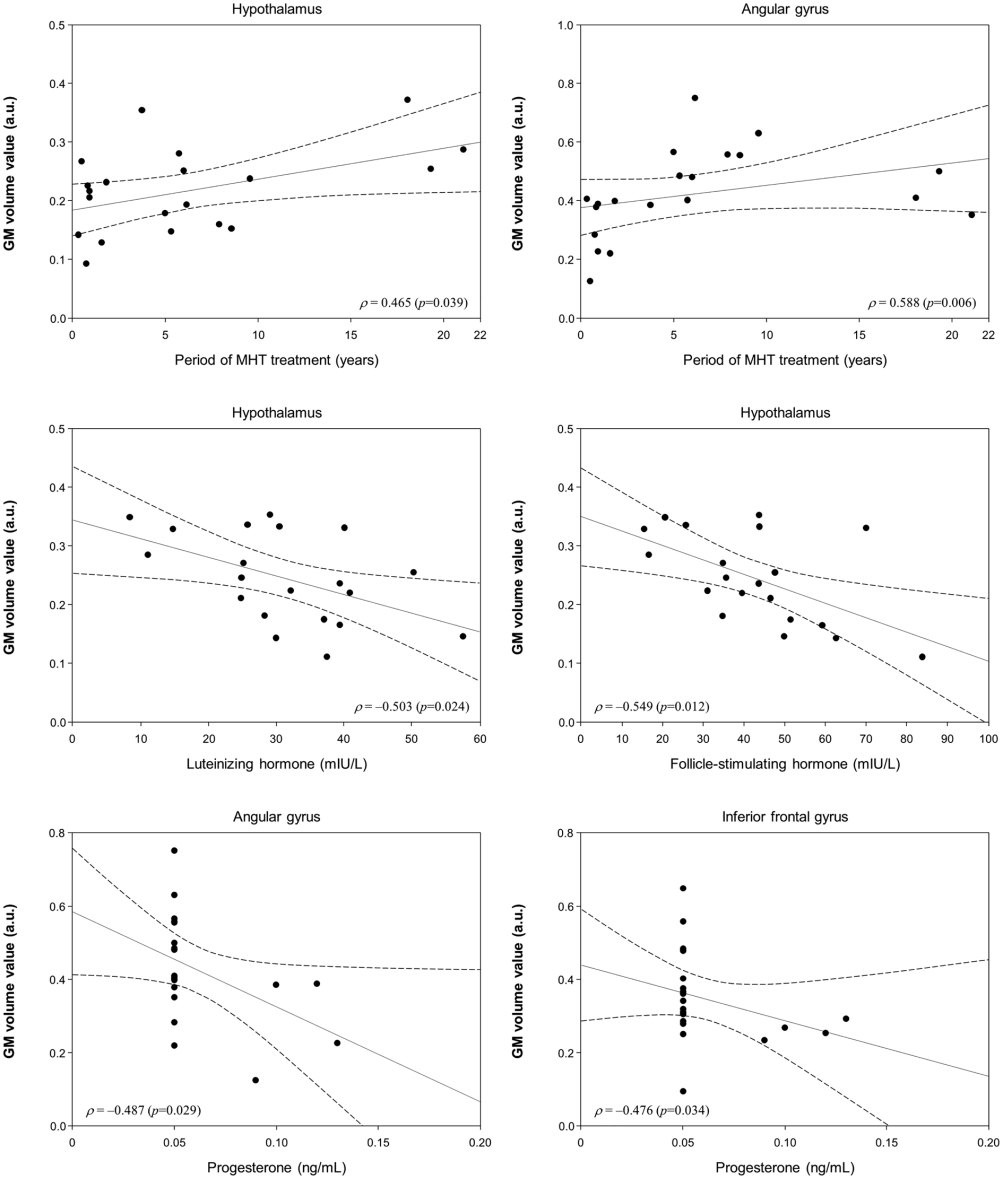
The period of MHT treatment in women was positively correlated with mean gray matter (GM) volume value in the hypothalamus and angular gyrus. Follicle-stimulating hormone (FSH), luteinizing hormone (LH) and progesterone (PG) levels in MHT women were negatively correlated with GM volume value in the hypothalamus, angular gyrus, and inferior frontal gyrus. Note that the y-axis scales (GM volume value) are different each other. Dotted lines show 95% confidence interval (CI). a.u. = arbitrary unit.

Figure 3 shows the correlation between cerebral volumes and MHT treatment. MHT women showed positive correlations between the treatment period and the volumes of the AnG (Spearman’s rho, ρ = 0.588; *p* = 0.006), and the treatment period and the volumes of the Hy (ρ = 0.465; *p* = 0.039), respectively. Meanwhile, MHT women showed negative correlations between FSH levels and the volumes of the Hy (ρ = − 0.549; *p* = 0.012), luteinizing hormone (LH) levels and the volumes of the Hy (ρ = − 0.503; *p* = 0.024), and the progesterone (PG) levels and the volumes of the IFG (ρ = − 0.476; *p* = 0.034) and the PG levels and the volumes of the AnG (ρ = − 0.487; *p* = 0.029).

## Discussion

This study showed that MHT menopausal women have greater cortical GM volumes than noMHT women within the same age range (mean, 57 years), which was positively correlated with MHT treatment. With DARTEL-based VBM, the volumes of subcortical structures in MHT women differed from those in noMHT women. In particular, localized GM volumes, including the SFG/MFG/IFG, Hy, ITG, PHG, Hi, Cb, PoG, PCu, AnG, MOs, SOG and PrG were larger in MHT women than those in noMHT women. Furthermore, MHT women showed positive correlations between treatment period and volumes in the AnG and Hy. The GM volume values of the Hy were negatively correlated with the levels of FSH and LH (menstrual cycle-related hormones associated with gonadal function). The GM volume values of the IFG and AnG were negatively correlated with PG levels in MHT women. However, using conventional volume measurement, there were no significant differences between the two groups in total intracranial volume.

Regarding the effect of hormone therapy, previous studies^25–27^ reported that estrogen increased the neutral effect or affected structural integrity in brain tissue, especially in the prefrontal, temporal, and parietal lobes. In the study, estradiol levels in pre-menopausal women were significantly higher than in post-menopausal women, while FSH was significantly lower. Estrogen production decreases with age during the transition to menopause; a higher level of estradiol positively affects cognitive function, neuroprotection, and associated brain functions^33^. ET may influence clinical outcomes through vascular changes or effects on regional brain volume, including neuronal architecture and synaptic density^26^. A morphological study^6^ evaluating the effect of menopause-related hormone in post-menopausal women reported that the reduced volumes observed in the superior temporal gyrus, IFG, olfactory cortex, and MOs are closely associated with menopause-related structural change. In our study, the mean period of MHT is approximately 6 years. The serum estradiol level in MHT women, such as pre-menopausal women, was higher than that of noMHT women, while the FSH level was lower. These hormonal changes in MTH women were similar to previous studies^25–27^, demonstrating level values before and after hormone therapy. Compared to noMHT women, the increments in local GM volume observed in MHT women could be associated with protection or suppression of cell loss or/and cell shrinkage related to MHT treatment.

Menopausal women with MHT showed larger GM volumes in key brain regions including of the SFG/MFG/IFG, Hy, ITG, PHG, Hi, Cb, PoG, PCu, AnG, MOs, SOG and PrG compared to noMHT women. ET as a MHT can modulate cell proliferation, differentiation, and survival in brain tissues^34^; moreover, estrogen treatment significantly increased the density of neurotransmitter serotonin-binding sites in the anterior frontal, cingulate, and olfactory cortex^35^. Estrogen receptors were ubiquitously localized in the frontal cortex; therefore, the SFG/MFG/IFG, and MOs may be capable of neuroprotection by MHT treatment. Additionally, MHT menopausal women showed larger GM volumes, including the PoG, PCu, and AnG in somatosensory cortices and the PrG and MOs in closely adjacent primary and secondary motor areas. There are differences in the somatosensory cortex by hormone therapy^36,37^. Khan et al*.*^37^ reported that estradiol enhanced rapid increases in dendritic spines in the somatosensory cortex, an area that contributed to working memory, as well as the prefrontal cortex. A previous study^38^ suggested that hormone therapy and estrogen exposure positively affect the motor system, thus counteracting aging-related reorganization. Thus, these volume changes might help improve the execution of the voluntary movement-related function and the receiving and processing of sensory information from throughout the body by MHT treatment. Moreover, hormone replacement therapy (HRT) was positively associated with better performance on tasks of verbal memory^39^. A positron emission tomography (PET) study showed evidence that ET (either with or without progestagen addition) modulates longitudinal changes in blood flow of the brain areas involved in cognitive function^40^. HRT group performing on tasks of verbal and visual memory demonstrated differences in the activation of regions subserving memory (especially the frontal lobe) compared to non-HRT control group^41^. Also, greater activation of the temporal gyrus, Hi and insula regions, and the PHG and IFG, was observed during verbal and visual memory tasks, respectively, in long-term HRT users (> 2 years) compared to non-HRT users^40^. Therefore, cerebral volume changes by MHT are closely associated with the alteration of brain blood flow and brain function.

Interestingly, MHT women showed greater hypothalamic volumes. The Hy, which is located in the center of the brain between the pituitary gland and the thalamus^42^, plays an important role in the production of hormones such as FSH, LH, dopamine, vasopressin, and gonadotropin-releasing hormone (GnRH); and helps to stimulate many important processes while maintaining the stable state of the body (homeostasis), such as body temperature, emotions, sleep cycles, sexual drive, blood pressure, and heart rate^43^. Especially in feedback mechanism for ovarian hormone production^44^, the secretion of female sex hormones starts with the secretion of a GnRH in the Hy of the brain. GnRH stimulates the pituitary to release two gonadotropins, FSH and LH. Released FSH and LH promote ovulation and stimulate secretion of the sex steroids estradiol (the most common estrogen, E2) and PG. Positive and negative feedback by E2 and PG acts on FSH and LH production as follows: high levels of E2 and PG stimulate FSH and LH secretion, and the low levels of E2 and PG cause negative feedback to suppress FSH and LH secretion. In present study, MHT-treated women had lower levels of FSH and LH than noMHT women (as Table 1), and their hypothalamic volume is negatively correlated with FSH and LH levels, as shown in Fig. 3. In old reproductive-age women as noMHT menopausal women, the patterns of hormone secretion with decreased ovarian function show significant alterations of hypothalamic-pituitary feedback mechanisms by menopause^43^. The study showed relative hypothalamic-pituitary insensitivity to estrogen in aging menopausal women manifested by positive and negative feedback mechanisms (from FSH and LH levels). In this study, both groups enrolled are age-matched and BMI-matched. Hypothalamic hormonal changes (lower FSH/LH and higher estradiol levels like premenopausal women) reflected the improvement in hypothalamic pituitary sensitivity by MHT treatment; therefore, these findings may support the idea that MHT treatment improves menopausal symptoms that disrupt life quality in menopausal women.

This study included several limitations. First, this study used DARTEL-based VBM method to analyze the GM difference between MHT and noMHT groups. The DARTEL method can be replaced by ‘Geodesic Shooting,’ which is supposedly more mathematically correct^45^. Further study will perform to clarify the difference between DARTEL and Geodesic Shooting methods for assessing GM difference associated with MHT. Second, sex hormones, aging, and metabolic status are important factors in brain volume changes in post-menopausal women that may interact. To overcome aging and metabolic status-related morphometric changes, we directly compared age-matched and BMI-matched post-menopausal women as the MHT and noMHT groups. Our study focused on GM volume in response to MHT; more studies are needed to evaluate changes in WM volume to understand the effect of MHT, particularly with the addition of diffusion tensor imaging, which might help assess MHT-related morphometric alterations. The information of other tissues such as WM, and CSF could be available for understanding MHT-related differences. Further study will perform to clarify WM and CSF difference by MHT. Third, MHT treatment plays a protective role in cognitive decline; however, we did not measure cognitive function using assessment tools, such as the Mini-Mental State Examination or other intelligence tests. Despite such limitations, we showed MHT-related morphometric differences in specific areas of the brain, suggesting a neuroprotective effect of hormone therapy in the cerebral cortex.

In conclusion, MHT-treated women might be larger GM volumes compared to menopausal women without MHT. The anatomical structures that showed greater volume in association with MHT included the deep brain areas (Hy, PHG, Hi), cerebellar cortex, frontal (SFG/MFG/IFG, MOs, PrG), inferior temporal, parietal (PoG, PCu, AnG), and superior occipital gyri. This study found a correlation between the levels of sex hormones and localized brain volumes following MHT. These findings would help us understand the interaction between brain volumes and levels of sex hormones following menopause and MHT.

## Materials and methods

### Study population

The study protocol was approved as a prospective research (2020–09-011–004) by the institutional review board of Wonkwang University Hospital according to clinical practice guidelines and all subjects gave written informed consent. A total of 41 individuals, 20 menopausal women with MHT (MHT group; mean age 57.2 ± 4.2 years) and age-matched 21 menopausal women without MHT (noMHT group; mean age: 56.6 ± 3.8 years), were enrolled in the Department of Obstetrics and Gynecology from January 2021 to December 2021. Both groups did not have a history of neurological and psychiatric disease. All participants were prescreened by physical and neurological examinations and interviewed by an obstetrician gynecologist. This study was conducted following the Helsinki Declaration and Good Clinical Practice guidelines.

All noMHT women were chosen according to the following criteria. First, a menopause diagnosis based on the stages of reproductive aging workshop + 10 and the regularity of menstrual bleeding. Second, FSH levels > 30 mIU/L. Third, more than 1 year since the last menstrual period. Fourth, no history of hysterectomy/bilateral oophorectomy or psychiatric/neurological illnesses. Fifth, no history of hormone and steroid treatment or oral contraception for 1 month before the study.

### Measurement of serum sex hormone levels

All participants underwent blood tests to compare circulating sex hormone levels between both groups. All MHT menopausal women had received oral administration for MHT of only estrogen or combination hormone replacement treatment as Premina, Angeliq, Livial, Progynova, and Duavive. The measured sex hormones were AMH, estradiol, testosterone (TS), FSH, LH, and progesterone (PG) (Table 1).

The levels of AMH, estradiol, FSH, LH, PG and TS were measured with chemiluminoimmunoassay (CLIA) on the UniCel DxI 800 (Beckman Coulter Inc., Fullerton, CA) and electrochemiluminescence immunoassay (ECLIA) on the Cobas e801 automatic analyzer (Roche Diagnostics GmbH, Mannheim, Germany). The following test kits were used: AMH Assay kit (AccuBind® ELISA & AccuLite® CLIA, USA), Estradiol II assay (Roche, Mannheim, Germany), Elecsys FSH (Roche, Mannheim, Germany), and Elecsys LH (Roche, Mannheim, Germany), Elecsys Progesterone III assay (Roche, Mannheim, Germany) and The Access Testosterone assay (Beckman Coulter Inc., Fullerton, CA). All sex hormone levels were expressed as mean (SEM).

### Magnetic resonance imaging (MRI)

MRI examinations were obtained using a 3 T MRI scanner (Ingenia Elition; Philips Healthcare, Best, The Netherlands) with a 32-channel adult head coil. Brain images were acquired using the 3-dimensional T1-weighted turbo field echo (3D T1W-TFE), and the scan parameters were as follows: TR/TE, 8.1/4.6 ms; FOV, 256 × 256 × 180 mm^3^; flip angle, 8°; slices, 180; voxel size, 1 × 1 × 1 mm^3^; acquisition time, 4 min 32 s.

### The structural image analysis procedure of VBM for data post-processing

The T1-weighted MRI data were post-processed using the statistical parametric mapping program (SPM 12; Wellcome Department of Imaging Neuroscience Group, University College London, London, United Kingdom) with DARTEL analysis.

VBM was used to evaluate differences in the localized volume of GM among the compared groups. The brain MR images were processed using an optimized protocol described in previous studies^32,46^. Before data processing, the brain images of the 41 women were aligned with an anterior–posterior commissure line. MR images were processed with nonuniformity correction to remove smoothly varying modulations of image intensity. The MRI data were segmented into GM, WM, and cerebrospinal fluid (CSF) using tissue probability maps from the SPM 12 templates (“gray.nii”, “white.nii”, and “csf.nii”, respectively). The images were normalized to the International Consortium for Brain Mapping template for East Asian brains and multiplied by the nonlinear components derived from the normalization matrix for modulation of GM volume. A mean template for GM was created using individual GM images. All the images were normalized to the Montreal Neurological Institute (MNI) template and were subsequently separated into GM images. All images were then smoothed with a 6-mm full width at the half-maximum isotropic Gaussian kernel to minimize cortical variation. TIV was measured by calculating the volumes of GM, WM, and CSF in each woman using conventional volume measurement. We used the general linear model and a two-sample test to examine voxel-wise GM differences between noMHT and MHT women in SPM.

Effect size for neuroimaging data is simply calculated as a Cohen’s d value^47^. For our given sample size (n = 41), the value d can be converted to a *t*-value (Cohen’s d = t/[n]^1/2^). The threshold *t*-value 3.0 is moderate effect size of Cohen’s d (0.5). A 2-sample *t-*test (*p* < 0.05, corrected family-wise error) and analysis of covariance (ANCOVA) adjusting for age and TIV were used to compare whole GM volume between noMHT and MHT women. The cluster size included more than 50 contiguous voxels. A correlation map was evaluated the regression between the levels of sexual hormones and mean GM volume in each brain area. Each brain area’s mask was applied to the evaluation of the mean GM volume value.

### Statistical analysis

All statistical analyzes were performed using the statistical package for the social sciences (SPSS ver.20; Chicago, Illinois, USA) and the SPM 12 program. Differences in sex hormone levels between the noMHT and MHT groups were analyzed using an independent two-sample *t*-test. The correlation between MHT (treatment period; levels of sex hormones) and brain volume variations were analyzed by simple regression in SPM and Spearman’s correlation in SPSS.

## Data Availability

The datasets used and/or analyzed during the current study available from the corresponding author on reasonable request.
